# Two-Dimensional Black Phosphorus Nanomaterials: Emerging Advances in Electrochemical Energy Storage Science

**DOI:** 10.1007/s40820-020-00510-5

**Published:** 2020-09-02

**Authors:** Junye Cheng, Lingfeng Gao, Tian Li, Shan Mei, Cong Wang, Bo Wen, Weichun Huang, Chao Li, Guangping Zheng, Hao Wang, Han Zhang

**Affiliations:** 1 Guangdong Provincial Key Laboratory of Micro/Nano Optomechatronics Engineering, College of Mechatronics and Control Engineering, Shenzhen University, Shenzhen, 518060 People’s Republic of China; 2 Collaborative Innovation Center for Optoelectronic Science and Technology, International Collaborative Laboratory of 2D Materials for Optoelectronic Science and Technology of Ministry of Education and Guangdong Province, Shenzhen University, Shenzhen, 518060 People’s Republic of China; 3 Department of Mechanical Engineering, Hong Kong Polytechnic University, Hung Hom, Kowloon, Hong Kong People’s Republic of China; 4 Department of Materials Science and Engineering, Drexel University, Philadelphia, PA 19104 USA; 5 Nantong Key Lab of Intelligent and New Energy Materials, College of Chemistry and Chemical Engineering, Nantong University, Nantong, 226019 Jiangsu People’s Republic of China

**Keywords:** 2D black phosphorus, Electronic structure, Supercapacitors, Batteries

## Abstract

Two-dimensional black phosphorus (2D BP), well known as phosphorene, has triggered tremendous attention since the first discovery in 2014. The unique puckered monolayer structure endows 2D BP intriguing properties, which facilitate its potential applications in various fields, such as catalyst, energy storage, sensor, etc. Owing to the large surface area, good electric conductivity, and high theoretical specific capacity, 2D BP has been widely studied as electrode materials and significantly enhanced the performance of energy storage devices. With the rapid development of energy storage devices based on 2D BP, a timely review on this topic is in demand to further extend the application of 2D BP in energy storage. In this review, recent advances in experimental and theoretical development of 2D BP are presented along with its structures, properties, and synthetic methods. Particularly, their emerging applications in electrochemical energy storage, including Li^−^/K^−^/Mg^−^/Na-ion, Li–S batteries, and supercapacitors, are systematically summarized with milestones as well as the challenges. Benefited from the fast-growing dynamic investigation of 2D BP, some possible improvements and constructive perspectives are provided to guide the design of 2D BP-based energy storage devices with high performance.

## Highlights

- Two-dimensional black phosphorus (2D BP) possesses huge potential in electrochemical energy storage field owing to its unique electronic structure, high charge carrier mobility, and large interlayer spacing.
- Comparison on the different preparation methods and processes, characteristics, and applications of few-layer BP is presented.
- The applications of 2D BP in electrochemical energy storage devices in these years are well reviewed.

## Graphical abstract

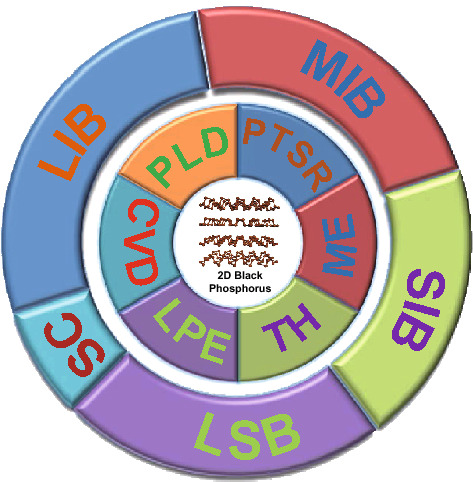

## Introduction

Two-dimensional (2D) materials have attracted intensive interests since the first discovery of single-layer graphene in 2004 [1]. Different from bulk graphite counterpart, graphene is constituted by a single-layer carbon atoms which are densely packed in a honeycomb crystal lattice [2]. Owing to the unique physical and chemical properties, graphene exhibits extremely large surface-to-volume ratio, quantum confinement [3–5], excellent electron mobility, thermal conductivity [6], strong mechanical properties [7], and high transparency [8]. Although these wonderful properties have allowed graphene to be applied in electronic field [9], the lack of bandgap has limited its performance. Consequently, other 2D materials have been explored to tackle the issues of graphene. Up to now, a group of 2D materials [10–13] have been intensively synthesized and investigated, including 2D black phosphorene (BP), hexagonal boron nitride (hBN), transition metal dichalcogenides (TMDs) [14], layered double hydroxides (LDHs), graphitic carbon nitride (g-C_3_N_4_) and transition-metal carbides/nitrides (MXene). Among them, BP is a rising-star 2D material, which has attracted numerous theoretical and experimental investigations because of the unique structure and intriguing anisotropic properties.

Phosphorus [15], in group V of the periodic table, is environmentally abundant (0.1% of the Earth’s crust) with four allotropes according to the atomic structure [16], including white, red [17], black, and purple phosphorus. Among these allotropes, BP is the most thermodynamically stable [18] and can be mutually transformed from white or red phosphorus. BP was first synthesized by Bridgman [19] under high pressure and temperature, but had been rarely studied during the twentieth century due to the extreme synthetic conditions [20]. Fortunately, Nilges et al. [21] successfully developed a method for the fabrication of BP in large scale through a chemical vapor transport approach, promoting the further property investigation of BP as well as its applications. In 2014, a monolayer of BP was successfully obtained by Zhang et al. [22] and Ye et al. [23] through a scotch-tape micro-cleavage approach. It is found that the 2D BP possesses puckered honeycomb structure with lattice constants of 4.58 (*a*) and 3.32 Å (*b*). Due to the similar translational structural symmetry and bond interactions, the bandgap of multilayer BP is similar to that of monolayer BP, indicating that the bandgap can be tuned by the layer thickness. Moreover, the bandgap remains direct at the Γ point of the Brillouin zone in a wide range of thickness, which means that the BP shows great potential to bridge the gap between zero-gap graphene and large-gap TMDs. In addition, 2D BP possesses a much larger specific surface area (~ 2630 m^2^ g^−1^) than graphene due to the puckered structure and increased layer distance. Therefore, 2D BP has been intensively studied and widely applied in various fields, such as ultrafast laser [24], bio-photonics [25], energy storage devices [26], optoelectronics [27], solar cell [28], and nanosensors [29].

Since the global warming and potential energy crisis in recent decades, environmentally friendly and sustainable technological products are getting more and more prevalent and vital. Therefore, novel products such as electric vehicles are becoming more demanding, which require high energy capacity and efficiency. Although conventional lithium-ion batteries have been widely spread out in human society, its performance does not meet the requirements for future development. Therefore, new electrode materials need to be developed as alternatives for conventional graphite-based electrode materials. Recent studies indicate that 2D BP and its bulk phase possess tunable direct bandgap [30], in-plane anisotropic structure and high charge carrier mobility [22, 23]. In addition, the theoretical specific capacity (~ 432.8 mAh g^−1^) of 2D BP is much higher than that of graphite (~ 372 mAh g^−1^) due to its larger layer spacing and folded structure. Owing to these features, 2D BP and its bulk phase show great energy storage ability both theoretically and experimentally [15, 31]. On the basis of the ultra-high performance, energy storage systems based on 2D BP and its bulk phase are feasible to be commercialized in the near future [21, 26, 32–34].

The intrinsic structure and unique properties of 2D BP have aroused great attentions in electrochemical energy storage, and notable progress has been achieved in recent 5 years. Thus, a timely review is in demand to further promote the development of 2D BP-based energy storage devices. In this review, the structural and physiochemical properties of BP are briefly summarized. Then, the recent experimental and theoretical progress in the preparation of bulk and 2D BP are systematically presented. Importantly, we focused on the latest advances in their practical applications, including lithium-/sodium-ion batteries and super-capacitors. Finally, exclusive insights in current challenges and future opportunities of 2D BP-based energy storage devices are provided, and novel design strategies are given for their future research directions.

## Properties of Black Phosphorus

### Atomic Structure

Similar as graphite, black phosphorus (BP) [35] is also a stacked-layer structured material with van der Waals interaction between different layers [36]. It has orthorhombic [37] unit cell consists of two layers with lattice constant of *a* = 0.45 nm, *b* = 0.34 nm and *c* = 1.12 nm (Table 1) [38–40]. The spacing between layers is 0.53 nm (0.33 nm for graphene) [41–43]. Layer distance along the channel is 0.308 nm, which is much larger than the 0.116 nm of cross-channel spacing. The distances between phosphorus atoms lying in the same plane and adjacent planes are 0.2224 and 0.2244 nm, respectively [32]. The significantly large interlayer distance indicates that BP is suitable as anode materials for hosting lithium (0.152 nm) and sodium (0.204 nm) ions.

**Table 1:** Detailed parameters of black phosphorus including lattice constant, interlayer distance, bond length, and density

Parameters	Values	Description	References
a	0.45 nm	Orthorhombic lattice constant	[37–40]
b	0.34 nm	Orthorhombic lattice constant	[37–40]
c	1.12 nm	Orthorhombic lattice constant	[37–40]
d1	0.53 nm	Maximum interlayer spacing	[41, 43]
d2	0.308 nm	Channel interlayer spacing	[41, 43]
d3	0.116 nm	Cross-channel interlayer spacing	[41, 43]
b1	0.2244 nm	Bond length in the same plane within one layer	[44]
b2	0.2244 nm	Bond length between adjacent plane within one layer	[44]
ρ	2.69 g cm^−3^	Single layer density	[47]

**Fig. 1:**
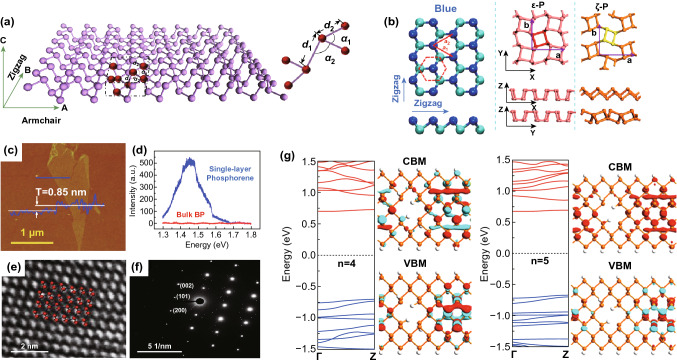
**a** Crystal structure of monolayer BP. The zigzag and armchair directions are represented by the green coordinates. The dash dot lines frame the unit cell of the BP; adapted with permission [44]. Copyright 2019 Elsevier. **b** Structures of three predicted polymorphs of phosphorene. Adapted with permission [45]. Copyright 2015 Nature Publishing Group. **c** AFM image; **d** Raman spectra of monolayer and bulk BP; reproduced with permission [23]. Copyright 2014, American Chemical Society; HR-TEM **e**, **f** SAED for few-layer 2D BP. Reproduced with permission [47]. Copyright 2015, American Chemical Society; **g** schematics of phosphorene nanoribbons model where n is the neck width and W is the nanoribbon width. Top two are zigzag nanoribbons with nanoholes in the middle and near the edge, while the bottom one is armchair nanoribbon. Computed bandgap structure for zigzag and armchair nanoribbons is shown on the top and bottom, respectively. Reproduced with permission [64]. Copyright 2020 Royal Society of Chemistry

As can be seen in Fig. 1a, phosphorene [44] is a single layer from its bulk phase and possesses a puckered honeycomb structure [45]. Atoms within each layer are covalently bonded in a triangular pyramid structure due to the *sp*^3^ hybridization, giving a density of 2.69 g cm^−3^ [46]. Along the horizontal direction, phosphorus atoms appear to have bilayer configuration in armchair direction, while zigzag configuration in the other direction. As revealed, the phosphorene monolayer (Fig. 1b) exhibits a honeycomb lattice structure with anisotropy along one basic vector, which can be ascribed to the non-planar structural ridges. The thickness of single-layer BP is ≈ 0.85 nm, which is larger than the theoretical value of 0.6 nm (Fig. 1c). The Raman spectrum of two-dimensional BP also shows thickness dependence (Fig. 1d) [23]. Unlike symmetrical graphene, photons, electrons, and phonons show highly anisotropic pattern in the anisotropic structure; hence, phosphorene shows great potential for the fabrication of thin-film electrodes and electronics. Figure 1f shows the selected electron diffraction pattern of phosphorene with the first three nearest reflexes in the [001] zone axis representing the (101), (002), and (200) planes, respectively [47–51]. This provides a standard approach to determine black phosphorus single crystal by X-ray diffraction. Indeed, complex facet information is observed in a black phosphorus film based on nanocrystals, e.g., (021), (040), and (117) planes [52–54]. This non-single crystal character can be ascribed to the rotational and vertical stacking of 2D materials or zone axis adjustments [55–59]. Furthermore, the relative intensity ratio between the (101) and (002) dots can be used to evaluate the layer number, for instance 2.6 for monolayer, 0 for bilayer, and 0.3 for trilayer phosphorene [47].

### Electronic Band Properties

Bearing a direct bandgap in nature, BP shows a reciprocal layer-dependent bandgap that varies from 0.3 to 1.5 eV when the thickness is reduced to single layer (phosphorene ~ 1.67 eV, two-layer BP ~ 1.08 eV, three-layer BP ~ 0.74 eV, BP ~ 0.4 eV) [60, 61]. Theoretical calculations have shown that the tunable bandgap is associated with the loss of interlayer hybridization in few layers system [62]. Therefore, a wide solar absorption become possible, and it allows BP to be applied in photovoltaic devices such as solar cell [28]. In addition to the layer thickness effect, its bandgap can be tuned by functional groups, strain [63, 64], rotation angles [65] between phosphorene sheets and electrical field. Sun et al. [64] theoretically investigated the electronic band properties of phosphorene nanoribbons by doping non-metallic elements (e.g., C, F, N, S, O, and Si) on the edge terminated with zigzag configuration. Phosphorene nanoribbons always show metallic behavior by doping with C, O, S, and Si, while it could behave like metal or semiconductor when zigzag edge is doped with H, F, or N. Moreover, enhancing externally applied transverse electric field could reduce the bandgap of phosphorene nanoribbons doped with H, F, and N effectively. Besides, bandgap also varies between phosphorene with edges terminated by armchair and zigzag configuration. When nanoholes are introduced to phosphorene nanoribbons, zigzag edge nanoribbons go through a direct-to-indirect bandgap transition, while armchair edge ones still possess their direct bandgap feature with increasing bandgap (Fig. 1g) [64]. Introducing nanoholes has little effect on structural stability, but the bandgap can thus be manipulated by applying transverse electric field or mechanical strain. The tunability of BP provides itself a large platform for applications [66–68].

### Electrical Performance

Bulk BP shows good electrical conductivity (300 S m^−1^) and is a typical p-type semiconductor [38, 39, 69]. Charge carrier mobility in semiconductor is an important parameter for electron and hole transportation with an inverse relationship to effective mass [70]. The electron and hole mobilities in bulk BP are 220 and 350 cm^2^ V^−1^ s^−1^, respectively [71]. Due to the in-plane anisotropy [72], effective electron and hole mass with regard to their mobility has been predicted by theoretical study. In the case of armchair and zigzag direction [73, 74], the effective masses are 0.17 and 1.12 m_o_ (1140 and 80 cm^2^ V^−1^ s^−1^) for electrons, while 0.15 and 6.35 m_o_ (700 and 26,000 cm^2^ V^−1^ s^−1^) for holes, respectively. The ultra-high mobility of holes proves the p-type semiconductor because of the extremely small potential even though the effective mass is significantly high.

### Optical Properties

As been mentioned, BP exhibits tunable direct-bandgap feature [75]. By manipulating layer number, the absorption rate of electromagnetic wave significantly increases and the absorption wavelength can be broadened to the full range of absorption spectrum [24, 25, 61, 76]. When number of layers is reduced to one, phosphorene shows a high absorption rate in the ultraviolet region [42]. Moreover, by applying 4% tensile strain, the optical bandgap of BP shifts to the entire visible range [15, 77], while a 4% compressive strain changes optical absorption to the infrared region [78]. Meanwhile, due to the in-plane anisotropy, direction of photon absorption can also affect the efficiency [40, 43]. Absorption coefficient in armchair direction is about 10 times larger than that along zigzag direction. Armchair direction also facilitates photon diffusion by 16 times compared to zigzag direction [41].

### Mechanical Properties

Besides the tunable bandgap, the mechanical properties of BP are also layer number dependent and anisotropic, which attracts a lot of attention. The Poisson’s ratio is an indication of anisotropic nature for two-dimensional BP. It was found to be 0.703 and 0.175 in the zigzag and armchair directions, respectively [79], while the minimum value of 0.064 was obtained along 47.5°. The excellent flexibility of thin-layer BP enables it to be highly stretched for flexible devices [80–83]. It has been theoretically proved that the phosphorene can reach a significant high tensile strain of 30% and 27% along armchair and zigzag direction [84]. Moreover, other theoretical calculations have shown the thermal and electrical properties can be associated with and modified by strain engineering [85, 86]. Young’s modulus and tensile strength are the most fundamental parameters to evaluate the stiffness of materials. The theoretically predicted young’s modulus of few-layer BP is 166 and 44 GPa, while the experimentally measured values are 60 and 30 GPa along the zigzag and armchair direction, respectively [87]. In general, an averaged young’s modulus of 94 GPa of all direction was obtained in 2D BP [88]. Therefore, 2D BP exhibits excellent flexibility due to the much smaller tensile strength compared with MoS_2_ (270 GPa) and graphene (1 TPa). This has embedded 2D BP with advantages to be applied into large-strain engineering application.

### Thermal Properties

Although BP is considered as the most thermodynamically stable state among different phosphorus allotropes, its 2D phase is highly unstable due to its hydrophilic characteristic, which makes it prone to oxidation. In addition, heating was also found to facilitate the degradation of BP. It was reported that the melting of thin-layer BP begin at 400 °C in vacuum and the decomposition temperature should be greater than that at around 450 °C [89]. The melting point of BP is between 600 and 1000 °C. Unfortunately, the exact melting point of thin-layer BP is still not clearly determined. The anisotropic nature of thermal conductivity has also received a lot of attention. For thin-layer BP, the thermal conductivity in zigzag direction is around 40 W m^−1^ K^−1^ [58], which is much higher than that in armchair direction [86]. As the thickness decreases, thermal conductivities along these two directions also significantly reduce and stop decreasing when layer thickness is below 15 nm [90]. This anisotropic thermal conductivity permits its application in both optoelectronic and thermoelectric devices [91].

## Synthesis Methods

**Fig. 2:**
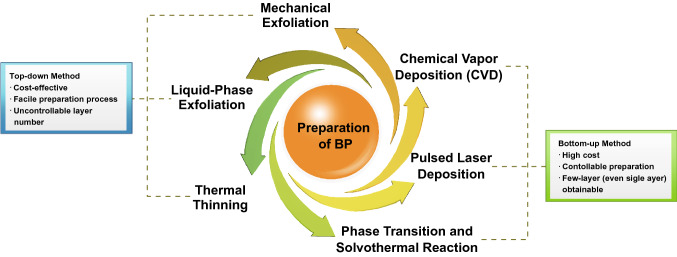
Overview of currently available preparation strategies and characteristics for fabricating 2D BP

In this section, different preparation methods for thin-layer BP will be discussed. From previous research work, it has been widely accepted that the quality of materials can be greatly affected by the reliability of synthesis methods. Generally, the preparation of thin-layer BP can be divided into two strategies: top-down (e.g., mechanical microcleavage and liquid phase exfoliation) and bottom-up (e.g. chemical vapor deposition and pulsed layer deposition) methods (Fig. 2). In top-down methods, thin-layer BP is usually obtained by breaking the van der Waals bonding among the stacked layers from its bulk phase. However, the bottom-up methods rely on the reaction (e.g., chemical) between precursors and directly synthesize the thin-layer BP.

### Top-Down Methods

#### Mechanical Cleavage

**Fig. 3:**
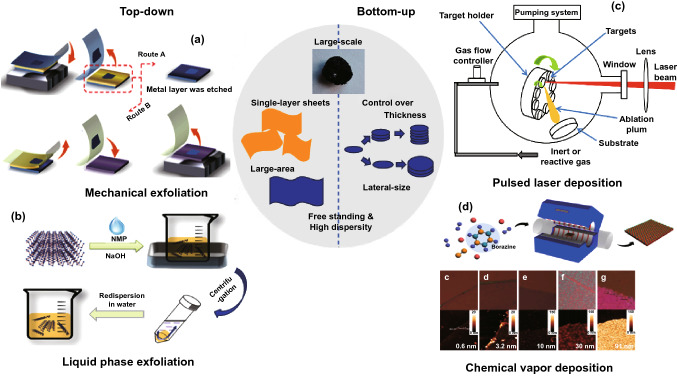
Schematic description of several typical top-down and bottom-up synthetic techniques of **a** mechanical cleavage; reproduced with permission [92]. Copyright 2018 Royal Society of Chemistry; **b** liquid phase exfoliation; adapted with permission [106]. Copyright 2015 Wiley Publishing Group; **c** pulsed laser deposition; adapted with permission [148]. Copyright 2018 Frontiers; **d** chemical vapor deposition. Reproduced with permission [135]. Copyright 2016 Nature Publishing Group

The mechanical exfoliation is the most conventional way to obtain the two-dimensional products from its bulk phase. In a typical process (Fig. 3a), the bulk BP is firstly stuck on a piece of scotch tape, while another piece of scotch tape is adhered on the other side of BP, followed by a peel-off action [92, 93]. The exfoliation energy of BP from multilevel quantum-chemical calculation is ~ 151 mV per atom [94, 95]. By repeating this process several times, thin-layer BP can then be obtained. Then, scotch tape residual needs to be removed by isopropanol, methanol, and acetone. The solvent residue is removed in a post-bake process under 180 °C. To avoid residuals from the scotch tape, a modified scotch tape method called all-dry transfer method has been developed. By using a thermal-release tape, samples with little residuals can be obtained with a high chance by controlling the peeling rate [96–100]. Although this method has been widely used in the laboratory, it does have a lot of limitations including uncontrollable size, thickness, and shape of the products. Besides, the low yield [22, 23] and poor repeatability restrict its scale-up production [100–105].

#### Liquid Phase Exfoliation

Compared with the mechanical exfoliation, liquid phase sonication exfoliation is considered as a controllable technique and it has already been widely applied for the fabrication of large quantities of 2D materials [106, 107]. To achieve the highest exfoliation rate [108], choosing the appropriate solvent becomes critical, and the optical spectroscopy has proved that the optimal solvent should have a surface tension of 35–40 mJ m^−2^ [109]. Solvents with surface tension within this range can minimize the costs of preparation and prohibit the restacking of BP thin layers [110]. To date, many solvents have been successfully used in liquid exfoliation of BP, including *N*-cyclohexyl-2-pyrrolidone (CHP), (*N*-methyl-2-pyrrolidone (NMP) [111, 112], dimethylformamide (DMF), acetone, ethanol, chloroform, hexane, 1,2-dichlorobenzene (DVB), tetrahydrofuran (THF), and dimethyl sulfoxide (DMSO) [113–116]. Among all these mentioned solvents, DMF and NMP have the optimal surface energy value [117], which are 37.1 and 40 mJ m^−2^, respectively [118]. Moreover, the use of organic solvents can protect the thin-layer BP surface against the oxidation from ambient environment. Sonication exfoliation process typically includes three steps which are immersion, probe/bath ultrasonication, and purification (Fig. 3b) [106, 119, 120]. In the second step, ultrasonic wave generates cavitation for bubbles and bubbles collapse between phosphorene layers with pressure released. Therefore, sonication time is one of the most important parameters, and the exfoliation efficiency increases with prolonged sonication time [121]. However, the yield in a sole sonication technique is quite low. Assisted sonication method has then been proposed with ionic intercalation [122], surfactant [118], microwave [123–125], electrochemical assistance [126]. The intercalation-assisted approach takes the advantages of significant volume expansion (e.g., 300%) when alkali metal ions are inserted into the 2D layered materials including graphene, etc. [125, 127]. During the volume expansion, the interlayer interaction is weakened, and the layers can thus be easily exfoliated by additional applied sonication [128]. The use of surfactant can help break the van der Waals interaction between phosphorene layers. One of the typical chemical surfactants that widely applied during sonication process is sodium dodecyl sulfate. Products from this approach have shown reliable and concentrated structure compared with mechanically exfoliated approach. Electrochemical-assisted sonication exfoliation has been proved to be facile, fast, and sustainable to acquire thin-layer BP [129, 130]. During this process, bulk BP acts as a working electrode (cathode) and platinum acts as the counter electrode (anode) in an electrolytic solution [131]. The system is connected to an external potentiostat power source and the applied voltage oxidizes water molecules, producing OH and O radicals. As electrons flow through the BP electrode, the radicals gather around the electrode and produce oxygen. The inter layer van der Waals interaction is thus weakened, causing structural deformation of the BP [132–134]. The effectiveness of this approach is highly dependent on the choice of electrolyte, bulk phase precursors, and operation voltage.

#### Thermal Thinning

It is well known that heat can facilitate the degradation of bulk BP in the ambient environment [18]. The bulk BP is firstly prepared and placed into a furnace with subsequent heating to around 400 °C in an argon atmosphere. By removing layer by layer, thin-layer BP with certain thickness can be obtained. This approach has a promise of large quantity of thin-layer BP in a relatively low cost.

### Bottom-Up Methods

#### Chemical Vapor Deposition (CVD)

**Fig. 4:**
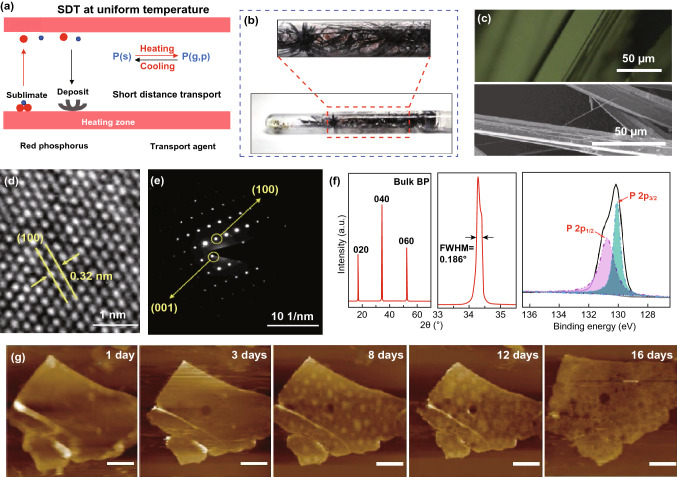
SDT growth of BP under a uniform temperature with the highest growth yield and purity. **a** Schematic mechanism of SDT technique. **b** Photographs of the products in a quartz ampoule after the reaction. **c** OM and SEM images of BP crystal laths; **d**, **e** HRTEM image and SAED pattern of a BP flake showing its high crystallinity; **f** XRD pattern of BP crystal. And magnified section of the (040) peak in the XRD pattern with a FWHM of 0.186^o^; **i** Raman spectrum of a BP crystal; **g** PL spectrum of monolayer BP under 77 K in vacuum with the sample was encapsulated by h-BN, showing a PL peak at 730.5 nm with a FWHM of 22.6 nm; **h** AFM images of a pristine BP flake with an as-exfoliated thickness of 25 nm, taken after ambient exposure for 1, 3, 8, 12, and 16 days. Reproduced with permission [142], Copyright 2020 Elsevier

CVD is the primarily used bottom-up method for manufacturing 2D materials of large size accompanied with high quality [135–138]. The early thin-layer BP was deposited on silicon oxide substrate with two steps similar to the vapor transport mineralization method (Fig. 3c) [135]. Firstly, RP thin film was put on a silicon oxide substrate in a vacuum chamber at 600 °C. Then, the thin-layer amorphous RP transformed into BP in the presence of SnI_4_/Sn with argon pressure at 2760 kPa, and few-layer (around 4) BP was then obtained. However, the thin-layer BP made through this early CVD method contains a lot of defects such as great amount of nanocrystalline with grain boundaries, which deteriorates its electrical and optical performance [139–141]. Unfortunately, the chemical vapor deposition of 2D BP still remains unexplored, and the current investigations in this field are quite limited. However, Liu et al. reported an efficient short-distance transport (SDT) growth approach to synthesize high-quality BP with high yield, and 98% of the RPRP can be converted to BP [142]. As can be seen in Fig. 4a, BP grows through a direct reaction of original materials, followed by a SDT process at high temperatures. Figure 4b shows the images of the ampoules after typical SDT growth. Apparently, a large quantity of BP can be obtained by the SDT process. Optical and scanning electron microscope (SEM) images clearly indicate that the BP crystal exhibits a lath-like morphology (Fig. 4c). In Fig. 4d, e, a typical HRTEM image and the corresponding SAED pattern indicate that the as-synthesized BP has an orthorhombic crystal structure [143]. In addition, the crystal structure of BP was further confirmed by X-ray diffraction (XRD, Fig. 4f). Each diffraction peak can be indexed to BP crystals with a preferred orientation of (0 k 0). The highest peak in the XRD pattern (Fig. 4f) is the (040) peak which shows a narrow full width with half maximum of 0.186^o^, indicating a high crystallinity [144]. As can be seen in the X-ray photoelectron spectroscopy (XPS, Fig. 4g), the P 2*p*_1/2_ and 2*p*_3/2_ core level peaks are located at 130.8 and 129.9 eV, respectively, which are in consistent with the previously results [145, 146]. The disappearance of the peak at 135.0 eV suggests that the as-grown BP has not been oxidized. AFM measurements were carried out to investigate the topographic features of the pristine BP flake (in laboratory light illumination (Fig. 4h) with humidity ranging from 70 to 91% and temperature ranging from 298 to 303 K), further confirming the effectiveness of this method. This work provides a new method to grow BP with tunable electronic structures and improved stability, which can be extended to these classes of materials in various areas.

#### Pulsed Laser Deposition

Pulsed laser deposition belongs to the physical vapor deposition (PVD) method. As shown in Fig. 3d, a high-power pulsed laser beam is targeted at the precursor material (RP), which needs to be sublimated in a vacuum chamber. The thin-layer BP film then grows on the substrate, which is driven by diffusion. This technique enables the fabrication of thin-layer BP with tunable sizes [147–149]. However, this method is not practical for massive production due to extremely high cost. Besides, thin-layer BP grown by this method has a structure with high entropy and relatively low charge carrier mobility, which is detrimental for its application in electronics.

#### Phase Transition and Solvothermal Reaction

**Fig. 5:**
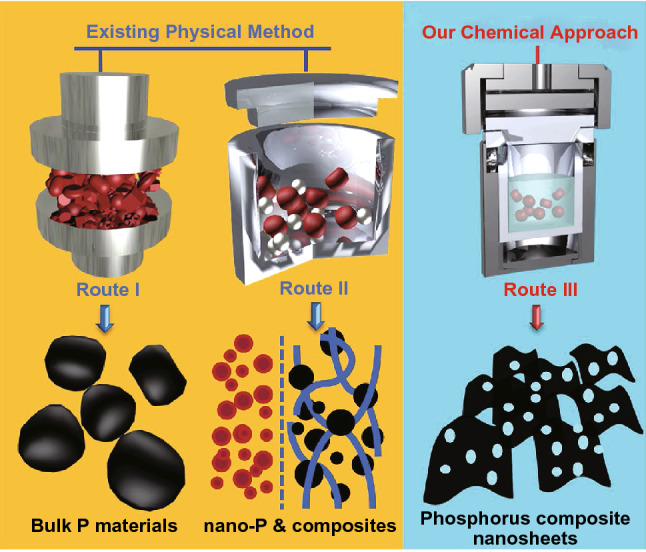
Schematic illustration of the formation of phosphorus-based materials. **a** Existing physical solid-state methods: Route (I) for the formation of bulk black P materials by traditional high-pressure method. Route (II) for the formation of nanosized (left) P material or its nanocomposites (right; carbon-, metal-composite, etc.) via normal/high energy mechanical milling methods. **b** Our chemical approach: Route (III) for the preparation of holey phosphorus-based composite nanosheets via a chemical solvothermal reaction. Reproduced with permission [151], Copyright 2016 Wiley Publishing Group

Recently, a phase transition approach for producing thin-layer BP on sapphire substrate with high crystallinity was reported. In this method, RP was firstly deposited on a sapphire substrate. Then, the RP thin film was transformed to thin-layer BP under high pressure and ambient temperature within several hours. The thin-layer BP film obtained by this technique is polycrystalline with grain size ranging from 40 to 70 microns [150]. Moreover, wet chemistry is also a common technique for 2D materials production. Solvothermal approach involves the iterative solid–vapor-solid process driven by evaporation–consolidation transformation of RP [151–158]. Recently, Zhang et al. demonstrated a sublimation-induced approach to prepare few-layer 2D holey phosphorus-based nanosheets from bulk RP under a wet-chemical solvothermal reaction. As shown in Fig. 5, the mechanism of this approach includes solid–vapor-solid transformation driven by continuous vaporization condensation process, as well as subsequent bottom-up assembly growth [151].

Up till now, the bottom-up method development is still at the initial stage with quite a lot of challenges to be solved. All reports related to the direct growth of thin-layer BP must start from RP, followed by phase transition. However, among all these bottom-up techniques, chemical vapor deposition can be potentially applicable to fabricate high-quality thin-layer BP since it has been applied to many other 2D materials over the past two decades. We also summarized the different techniques and make a comparison on preparation method and process, thickness, characteristics, and application of few-layer BP (Table 2).

**Table 2:** Summary of the comparison on the different preparation method and process, thickness, characteristics, and application of few-layer BP

Techniques	Classification	Methods	Solvent	BP thickness	Lateral control	Characteristics	Applications	References
*Top*-*down*								
Mechanical exfoliation	Top-down	Sticky-tape		< 7.5 nm	Lack of control	High carrier mobility with low production yield limit	FET	[80–94]
Sonication liquid exfoliation	Top-down		NMP	3–5 Layers	Lack of control	High yield	Semiconductor device	[95–121]
			DMF & DMSO	5–20 nm		Highly crystalline	Electronic device	
			CHP	8–11 Layers		Stable	Ultrafast saturable absorbers, gas sensors	
Thermal thinning	Top-down				Controllable	Potential for massive fabrication due to low cost		[18]
*Bottom*-*up*								
Chemical vapor deposition	Bottom-up	CVD		4 Layers	Controllable	Numerous defects introduced Large area potential for massive production	FET	[122–128]
Pulsed laser deposition	Bottom-up				Controllable	Flexible size with limited carrier mobility due to disordered structure		[129]
Phase transition and solvothermal reaction	Bottom-up	Mineralizer assisted (phase transition) Sublimation induced (solvothermal)	Ethanol (solvothermal)	< 5 nm (solvothermal)	Lack of control	High on/off ratios (phase transition) Holey morphology (solvothermal)	Optoelectronic device (phase transition) Batteries (solvothermal)	[130–136, 147]

## Energy Storage Applications

Rechargeable/secondary alkali ion batteries, especially Li-ion batteries, are popular due to its high energy density [159–161], good cycling stability [162, 163], and fast charging rate [164]. Li-ion batteries have already been applied in many electronic devices including laptops, mobile phones, vehicles, and vessels. Lithium is widely used because of its lightest weight in metals, lowest redox potential (− 3.04 V vs SHE, compared with − 2.93 and − 2.71 V for potassium and sodium), and lowest metallic atom radius [165]. A typical rechargeable battery generally includes anode, cathode, electrolyte, and membrane separator. Anode and cathode act as the host of the alkali ions diffusing in the electrolyte with a membrane separator preventing short circuit. The energy density and charging rate are determined by the cathodic and anodic specific capacity. However, the development of current commercialized batteries has come to a bottleneck. Therefore, a novel designed battery with significantly improved energy density and charging rate accompanied with high reliability is highly demanding to meet future requirements.

The working mechanism of secondary batteries will be discussed first [166]. During a discharge process, the potential difference between anode and cathode initiates the alkali atoms, which are intercalated in the anode materials, donating their valance electrons to the external circuit, and transforming to alkali cations. As the anode continuously losing alkali atoms, the potential difference gradually reduces to zero. Those donated electrons eventually gathered at the cathode, drawing those alkali cations through the membrane and accumulate in cathode materials. During a charging process, an external power source is connected to the battery, taking away alkali atoms electrons and forced alkali cations to diffuse toward the anode. These atoms are then intercalated in the anode (e.g., graphite), and the energy is stored in such a manner.

To improve the performance of current secondary batteries, investigating new anode material that has high capacity is an important aspect. Anode materials play a significant role in Li-ion battery because lithium ions have different intercalation behaviors in different materials, which greatly affect the overall capacity of the battery. Currently, graphite is the most widely used material, while other 2D materials such as graphene, MXene, metal oxides have also been deeply investigated. However, they all have their own disadvantages preventing them from applications. Thus, new anode material development is still an important topic in battery research.

**Fig. 6:**
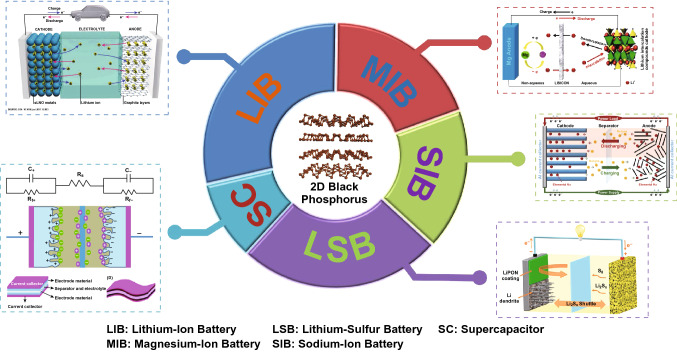
A summary of the potential applications of 2D BP. The pie chart shows the ratio of publications in each explored application of 2D BP with respect to the total number of publications. LIB image: reprinted with permission from ref [173], Copyright 2018 Elsevier; MIB image: reprinted with permission from ref [171], Copyright 2015 Nature Publishing Group; SIB image: reprinted with permission from ref [202], Copyright 2015 Elsevier; LSB image: reprinted with permission from Ref. [178], Copyright 2019 Elsevier; SC image: reprinted with permission from Ref. [227], Copyright 2014 Royal Society of Chemistry

Recently, BP has been intensively studied as an anode material and is considered to be a potential alternative for conventional anode materials. The bulk phosphorus material shows high energy capacity [166–168] (2600 mAh g^−1^) with low diffusion energy barrier (0.035 eV and 0.064 eV for Li and Na batteries, respectively) theoretically [165]. During charging, the alkali atoms intercalate into BP layers along zigzag direction accompanied by subsequent expansion along armchair direction because of the shifting from layers. However, the enormously large volume expansion (~ 300%) during discharging leads to a poor cycling performance, which limits its application [169]. Meanwhile, its 2D structure has already been predicted to have relatively high performance in energy storage application from theoretical calculation, while volume expansion can be suppressed. Therefore, in-depth review of the application of thin-layer BP in energy storage is valuable to reveal its advantages and limitations for future design. Figure 6 summarizes the applications of 2D BP, and the pie chart shows the ratio of publications with respect to the total number of publications. Owing to the fascinating electronic properties of 2D BP and large specific area, BP is widely used in electrochemical energy storage devices.

### Lithium-Ion Batteries (LIBs)

#### Thin-Layer BP as Anode

Rechargeable Li-ion batteries with excellent cycling stability [158, 170–173], high storage capacity and energy density [160, 168, 174–177] are the dominant energy storage units for consumer electronics [178–180] such as smartphones and tablets [177]. Based above merits, Zhao et al. [181] calculated the energy storage capability of phosphorene in Li-ion batteries through first-principle methods. It was found that phosphorene, as anode materials, could reach a high specific capacity of 432.79 mAh g^−1^, which is better than graphite (372 mAh g^−1^) and Ti_3_C_2_ (320 mAh g^−1^). It was also found that the binding energy of lithium atoms with phosphorene (2.16 eV) is much larger than that of other 2D materials (e.g., graphene 1.04 eV, MoS_2_ 2.12 eV), which implied that the lithium atoms can be stably adsorbed by phosphorene [182–184]. Interestingly, the binding energy further increases when defects are introduced to phosphorene. For example, lithium atoms are inclined to occupy the sites near a vacancy in phosphorene, with binding energy as high as 3.31 eV [34]. Moreover, the puckered surface of phosphorene can provide large surface area for lithium-ion insertion, and the diffusion energy barrier in the zigzag and armchair directions is anisotropic. It has been calculated that the diffusion energy barrier along zigzag direction (0.08 eV) is much smaller than graphene (0.327 eV) and MoS_2_ (0.25 eV), and the diffusion rate under ambient condition is 100 and 10,000 times higher than MoS_2_ and graphene [182, 185]. By introducing vacancies between adjacent grooves, lithium atoms will diffuse between channels in the armchair direction with relatively high diffusion energy barrier of 0.13 eV. The average voltage estimated for lithium atoms alloy is around 2.9 V, which is applicable for high-voltage applications [183]. During the lithiation, the semiconducting to conducting transition of black phosphorene allows it to be used as electrode. Besides that, the mechanical properties of phosphorene can be improved due to the lack of van der Waals interaction, and its 2D structure can be maintained during de-lithiation process with negligible volume change (~ 0.2%) [181].

**Fig. 7:**
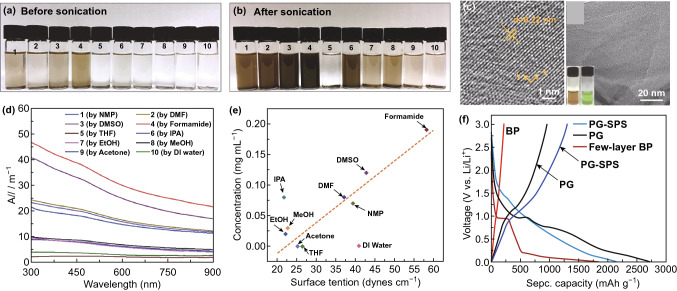
**a** Liquid exfoliation of black phosphorus in a series of organic solvents before sonication. **b** Solution containing 2D black phosphorus stored 12 h after sonication. **c** HRTEM and TEM images of black phosphorus collected after centrifugation at 2000 and 400 rpm lying on the left and right side, respectively. Insets are the pics (brown) and Tyndall effect (green) of the BP at 4000 rpm dispersions in formamide. **d** Optical absorption spectrum of minor dispersions in ethanol. **e** Surface tension of solution containing black phosphorus with different concentration in a different organic solvent. **f** First cycle of galvanic charge/discharge performance of thin-layer BP, 2D BP/graphene and 2D BP/graphene with spark pulsed sintering at a current density of 100 mA g^−1^. Adapted with permission [187]. Copyright 2016 John Wiley and Sons. (Color figure online)

Compared with single-layer phosphorene, few-layer BP can be also considered as a potential candidate for anode material in lithium-ion batteries. DFT calculations have shown that the diffusion energy barrier for double-layer BP is 0.72 eV, which is slightly lower than phosphorene (0.76 eV) [186]. Also, the binding energy of lithium atoms in the double-layer BP is 3.1 eV, compared to 2.16 eV in phosphorene. It indicates that lithium atoms bind more strongly with double-layer BP, which can endow it with a lower capacity but a higher voltage. At the same time, when Li ion battery with thin-layer BP anode is fully charged, the minimal distance between lithium atoms is 0.306 nm and 0.331 nm for phosphorene and double-layer BP, respectively [186]. Both are larger than the bonding distance of lithium dimers (0.274 nm). Therefore, the formation of lithium dendrite can be significantly suppressed due to the long Li–Li distance, assuring a good cycling performance of the Li ion battery. Zhang et al. [187] fabricated thin-layer BP by liquid exfoliation and used it as anode material for lithium-ion battery (Fig. 7). The battery exhibited a reversible specific capacity of 210 mAh g^−1^, but the columbic efficiency was only around 11.5%, which is very low. This inferior performance could be caused by the side reaction during lithiation process.

2D BP has showed great potential as LIB electrodes, but it also experienced several serious weaknesses. For instance, BP sheets are liable to self-stacking due to the van der Waals force between each other. In addition, due to the large specific surface area (SSA) of 2D BP, additional electrolytes will be consumed in the first cycle to generate solid electrolyte interphase layer, resulting in a large initial irreversible capacity, while Columbic efficiency is low. Higher SSA also leads to an adverse parasitic reaction between 2D BP and electrolytes, which further results in poor cycle life and potential safety issues. Generally, because of the low tap density of two-dimensional electrode material, the volume energy density of two-dimensional electrode is low. The construction and intercalation of heterogeneous structures can effectively solve these problems. Drawing inspiration from engineered graphene-based materials is another effective way to achieve high-quality 2D BP for LIBs.

#### Thin-Layer Black Phosphorus Composite as Anode

Although thin-layer phosphorene has the potential serving as anode materials, some challenges still exit that prohibit its applications. Poor stability in ambient environment is the primary obstacle, which has been observed in many preparation techniques [10, 109]. It has been revealed that oxygen, water, and visible light are the main three factors that cause the degradation of phosphorene [44, 92, 103, 115, 188–192]. First, the O_2_^−^ is generated in a redox reaction under ambient light on the surface of phosphorene. Then, it detaches from the surface and form covalent bonding with phosphorus atoms. Finally, the water molecules draw out the oxygen atoms from the surface, and the previous bonded phosphorus atoms were removed from the phosphorene surface through hydrogen bond. This degradation process results the decomposition of phosphorene and exposes the next layer for oxidation [193]. In general, light is found to be the main governing parameter of phosphorene degradation, which can be explained by Eqs. (1) and (2):

1 $${\text{O}}_{2}  + hv \to {\text{O}}_{2}^{ - }  + h^{ + }  $$

2 $$ {\text{O}}_{2}^{ - } + {\text{P}} + h^{ + } \to P_{n} O_{m} $$

Due to its enormous surface area, thin-layer phosphorus has a low coulombic efficient from the parasitic reaction with electrolyte. Besides, the energy density may be deteriorated due to the low packing efficiency. Moreover, 2D BP can easily cluster and restack layer by layer, and phosphorene may eventually lose its 2D characteristics. Therefore, composites based on thin-layer BP were proposed to resolve all the problems mentioned above.

**Fig. 8:**
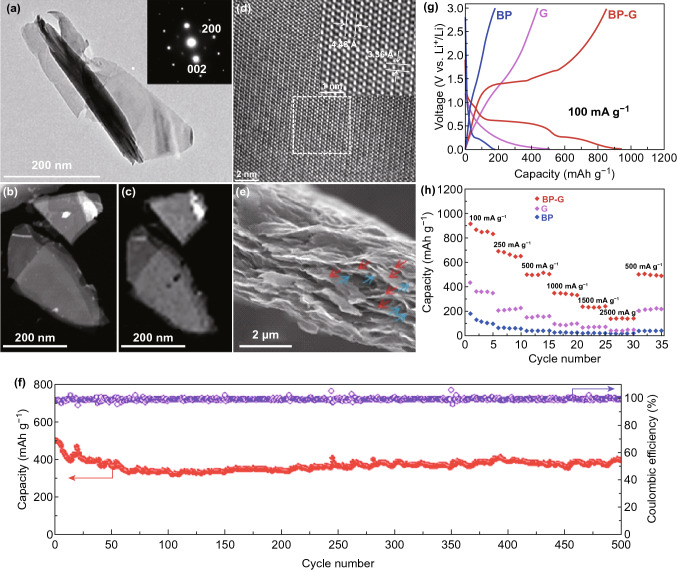
**a** TEM image of BP nanosheets with the zone axis along [010] in electron diffraction pattern. **b** STEM-HAADF image of two thin-layer black phosphorus. **c** EDS mapping of elements **d** HRTEM images of thin-layer black phosphorus **e** SEM images of 2D BP/graphene composite materials cross-sectional view. **f** Second galvanic charge/discharge performance of thin-layer BP, graphene and 2D BP/graphene hybrid sheets at a current density of 100 mA g^−1^. **g** Rate performance of thin-layer black phosphorus, graphene and 2D BP/graphene hybrid sheets at different current densities. **h** Cycling performance and coulombic efficiency of 2D BP/graphene composite anode materials under a current density of 500 mA g^−1^ after 500 cycles. Adapted with permission [137]. Copyright 2016 John Wiley and Sons

**Fig. 9:**
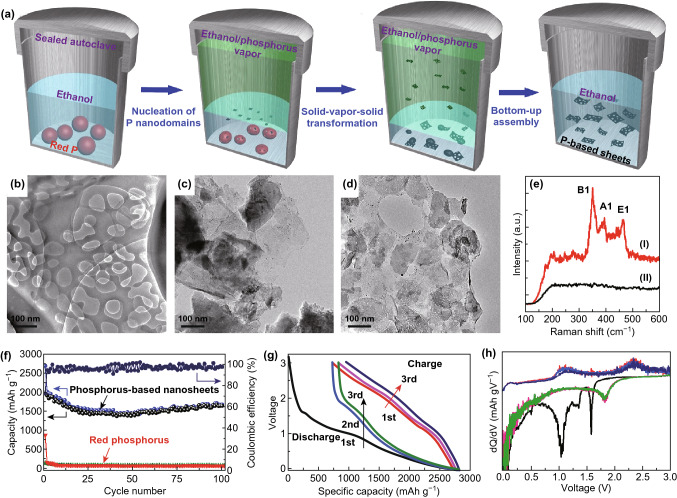
**a** Formation schematics of the holey phosphorus composite nanosheets. The morphology evolution of the bulk red phosphorus materials during the high-temperature solvothermal reaction at different reaction time of **b** 2 h, **c** 12 h, and **d** 24 h. **e** Raman spectra of (I) the precursor and (II) the final products. **f** Cycle performance comparison of phosphorus composites nanosheets and red phosphorus materials at a current density of 0.2 A g^−1^. **g** Voltage profiles of phosphorus composite nanosheets at 0.2 A g^−1^. **h** is the associated derivative − d*Q*/d*V* plot from **g**. In **h**, a continuous reduction potential is observed below 1.0 V (with the oxidation peak at ≈ 0.1, 1.1 V) and a stable reduction peak at ≈ 1.8 V (with oxidation peak at 2.3 V) are observed for phosphorus composite nanosheet samples. Reproduced with permission [151], Copyright 2016 Wiley Publishing Group

Guo et al. [183] studied the composite materials based on phosphorene and graphene, which was used as anode through first-principle calculation. Compared to mono-phosphorene/graphene [186], such composite design exhibits better stiffness, and energy specific capacity, and fast diffusion capability, conductivity, and diffusion rate of lithium atoms. This can be attributed to the following reasons: (1) elastic buffer spacing for volume expansion offered by flexible graphene layer; (2) enhanced conductivity; (3) improved stability of phosphorene by avoiding distortion of phosphorene after lithiation process. Zhang et al. [187] reported thin-layer BP/graphene with improved first-cycle coulombic efficiency of 34.3% (~ 11.5% in phosphorene). Under a current density of 100 mAh g^−1^, the energy specific capacity can reach as high as 820 mAh g^−1^. As can be seen in Fig. 8, Chen et al. [137] developed a paper-like flexible thin-layer BP/graphene anode material, which delivers a specific energy capacity of 920 mAh g^−1^ under a current density of 100 mA g^−1^. It can maintain 80.2% of its capacity, and the coulombic efficiency fluctuates near 100% after 500 cycles under a current density of 500 mA g^−1^. In order to pursue higher capacity and rate capacity in Li-ion batteries, a sublimation-induced strategy has been developed to fabricate phosphorus-based composite nanosheets by a chemistry-based solvothermal reaction (Fig. 9a) [151]. Porous structure generated from the RP precursor was observed in the TEM images (Fig. 9b–d), accompanying with trace amount of nanosheets at the short reaction time. The pores were well distributed with an average size smaller than 100 nm. Raman spectrum further confirms the transformation from RP precursors to phosphorus composited nanosheets (Fig. 9e). More importantly, it was observed that the discharge capacity maintained at 1683 mAh g^−1^ after 100 cycles (Fig. 9f). Unlike the crystalline phosphorus materials with a steady reaction plateau, the redox of these phosphorus-based nanosheets exhibits a continuous slope profile with slightly higher working potential (Fig. 9g, h). The rate performance was evaluated to confirm the potential of employing the phosphorus composite nanosheet electrode in LIB at high current density. The discharge capacity decreased from 1600 to 630 mAh g^−1^as the current density increasing from 0.5 to 20 A g^−1^, indicating the best performance for the phosphorus nanostructures or composite anode for LIB at high scan rates. Particularly, the nanopores were large enough to act as lithium-ion reservoirs for neighboring layers, which speeded up the ion transport and facilitated ion access to the entire surface. It can be seen that 2D BP composite, as a positive electrode, can effectively overcome the deficiency of single BP and has a great prospect in LIBs.

### Sodium-Ion Batteries (SIBs) and Beyond

**Fig. 10:**
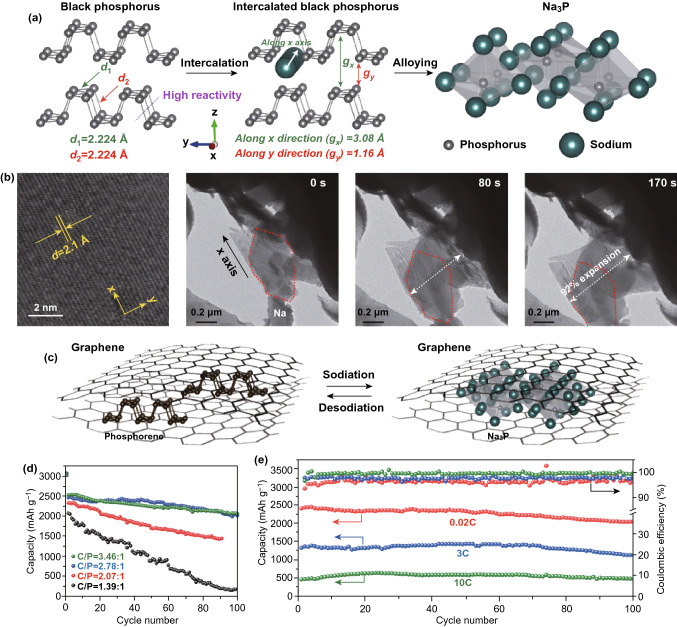
a Schematics of black phosphorus before sodiation, during intercalation and after alloying. **b** High-resolution and bright-field of TEM images of black phosphorus intercalation along the zigzag direction while volume expansion along the armchair direction. **c** Schematics of 2D black phosphorus/graphene composite materials before and after sodiation. **d** Cycling performance of 2D BP/graphene composite anode materials with different mole ratios after 100 cycles at a current density of 50 mA g^−1^. **e** Cycling performance and coulombic efficiency of 2D BP/graphene (48.3 wt%) after 100 cycles at different current densities. Adapted with permission [204]. Copyright 2015 Nature

**Fig. 11:**
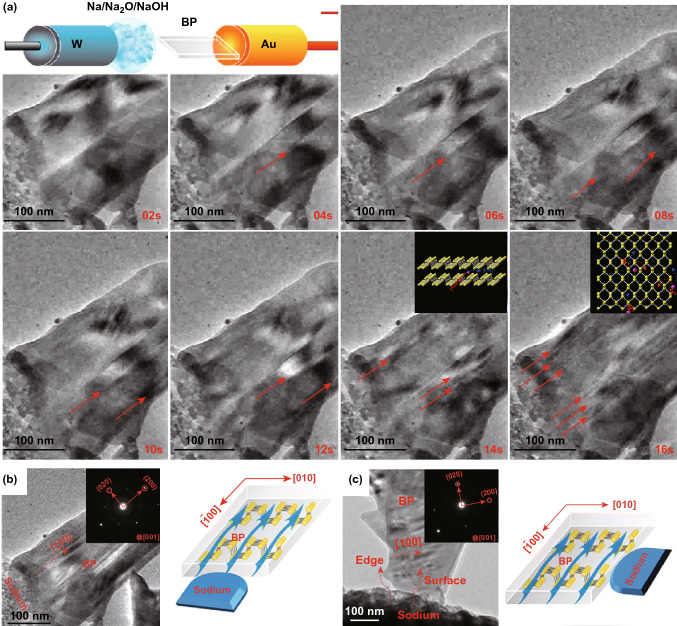
**a** In situ TEM snapshots showing the structure evolution of a few-layer phosphorene nanoflake during the sodium transport process. Images are taken during the first 24 s of sodium-ion transport process. The red arrows indicate the position of the formed stripes. The inset is the schematic of the experimental setups. Insets show schematic of sodium-ion transport in interlayer of few-layer phosphorene. Phosphorus and sodium atoms are shown in yellow and purple, respectively. Energy profiles for sodium-ion diffusion in different directions in few-layer phosphorene. **b, c** Sodium transport in few-layer phosphorene with different contact geometries with respect to the sodium source. **b** Contact interface normal to the [100] direction. The inset shows corresponding electron diffraction pattern of the few-layer phosphorene in panel **a**. **c** Contact interface parallel to the [100] direction. The inset shows the corresponding SAED pattern of the few-layer phosphorene in panel **b**. Adapted with permission [205]. Copyright 2016 American Chemical Society

Compared to lithium-ion batteries, sodium-ion batteries have attracted more attentions owing to the low cost and natural abundance of sodium element. Sodium can be also used to replace copper as the anodic current collector due to the lack of alloying with aluminum under a relatively low voltage [164, 194–202]. It has been considered as promising candidate to replace the conventional batteries in future. The charging and discharging mechanism is almost identical with conventional lithium-ion batteries. The sodium atoms are stagnated between electrodes interlayer in electrolyte during sodiation and de-sodiation processes. At a low sodium concentration, the intercalation process proceeds until it starts alloying process at a high sodium concentration. The potential is generated between the difference of sodium atoms that are stored in anode and cathode. However, the atomic radius of sodium atom is larger than that of lithium. Such a difference makes contributions to large volume change during a relatively slow electrochemical interaction. Previous theoretical studies have shown that thin-layer BP can be applied as anode materials in sodium-ion batteries. Kulish et al. [203] provided an ab initio calculation and revealed that the energy barrier for the diffusion of sodium ions along the zigzag direction is as small as 0.04 eV. Similar to lithium ions, the strong binding energy between phosphorus and sodium atoms indicates that sodium atoms can be stored without forming cluster sites. Phosphorene exhibits a high theoretical capacity resulting from its strong absorption ability of sodium atoms by forming NaP and Na_2_P (433 and 865 mAh g^−1^). Cui et al. [204] fabricated the first sodium-ion batteries with anode based on sandwich-like structured phosphorene/graphene composite (Fig. 10). A high reversible specific capacity of 2440 mAh g^−1^ under current density at 0.02 C was achieved. After 100 cycles, the sodium-ion batteries can keep around 85% (2080 mAh g^−1^) of the specific capacity under the same current density (Fig. 10d). A similar approach is replacing the graphene by sandwich-like *h*-BN nanosheets. When this composite was employed as anode for sodium-ion batteries, the performance has been significantly improved. The reversible capacity can reach as high as lithium-ion batteries with a stable electron transfer mechanism. Furthermore, adjusting the mass ratio between 2D materials and BP is important to reach the optimum performance. This approach is a promising technique resulting from its high theoretical specific capacity and low diffusion barrier energy. Recently, thin-layer BP with different thicknesses (2–11) was studied as anode material for sodium-ion batteries. High specific capacity of 1968 mAh g^−1^ at a current density of 100 mA g^−1^ was achieved [130]. For learning about the interior mechanism in the sodium battery, Nie et al. [205] demonstrated in situ TEM and complementary DFT simulations, which can be used to unveil the migration pathways of sodium ions inside the phosphorene. Due to the highly anisotropic diffusion property, the early stage of sodiation can be associated with the transportation of sodium ions along the [100] direction (Fig. 11a). Additionally, a faster diffusion of the sodium ion was achieved due to the zigzag edge of the phosphorene (Fig. 11b, c). These experimental and theoretical investigations provide a significant understanding of ionic transport properties of phosphorene, which also guideline the design of optimal electrode with high performance. Since the sodium ion prefers to diffuse along the [100] direction, minimizing the dimension of the phosphorene would provide facile diffusion channels, improving the performance of the phosphorene nanoribbons-based sodium-ion batteries. Fonsaca’s group and Guo et al. also demonstrated the 2D BP-based hybrid materials could serve as excellent SIB electrodes with good electrochemical cycling stability [200, 201].

**Fig. 12:**
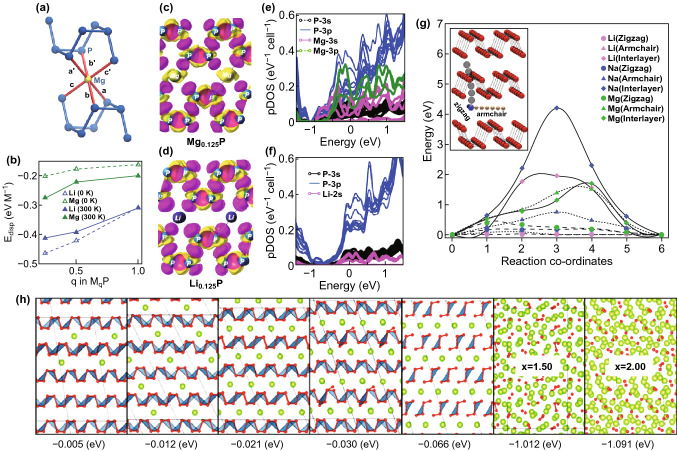
**a** Octahedral C.N. of Mg-ions at the a-phase, **b** the dispersive interaction per M-ions at a (M0.125P), **b** (M0.50P), and g (MP) phases. Differential charge density for M0.125P (M = Li/Mg), while intercalated M is at the transition state during diffusion along the x-channel (side view): **c** M = Li and **d** M = Mg. The loss of electrons is indicated in yellow and the gain of electrons is indicated in pink. The projected density of states (pDOS) of M0.125P (TR): **e** for M = Li and **f** for M = Mg. Adapted with permission [210]. Copyright 2016 Royal Society of Chemistry. **g** Diffusion barriers of Li, Na and Mg in black phosphorus along three different diffusion paths. Color codes are pink = Li, blue = Na, and green = Mg. **h** Insertion mechanism in black phosphorus with an increase in concentration of Mg. The solid gray line represents a supercell containing 64 P atoms, and the number represents the formation energy. Adapted with permission [211]. Copyright 2016 Royal Society of Chemistry. (Color figure online)

Magnesium is another viable alternative to replace lithium and sodium [171]. Low cost, natural abundance, advantages qualities and low reduction potential of magnesium (− 2.37 V) make it an ideal anode material for secondary batteries [206, 207]. Magnesium ions have a low diffusion barrier energy along zigzag and armchair direction [208, 209]. Thin-layer phosphorus can maintain its structure by forming Mg_0.5_P after been alloyed. During discharging, the magnesium atoms provide two valence electrons per atom. The theoretical volume and specific capacity of magnesium-ion batteries are 3833 mAh ^−3^ and 2205 mAh g^−1^, respectively. Banerjee et al. [210] presented the synergistic interaction between phosphorus and magnesium atoms, which dramatically reduced the diffusion barrier and enhanced the anodic voltage. By density function theory calculation (Fig. 12a), Hembram et al. [211] studied the magnesiation process in atomistic simulation and analyzed through first-principle calculations (Fig. 12b). It is revealed that phosphorene can be applied as anode because it can alloy with magnesium atoms by forming Mg_2_P (Fig. 12c). Jin et al. [209] found that the absorption energy is about − 1.09 eV for absorption of magnesium atoms on phosphorene. All the theoretical studies and experimental investigations have shown that thin-layer BP is an ideal anode material for magnesium-ion batteries. Recently, it has been proved by simulation and calculation that BP-based anodes show promising energy storage capabilities in potassium ion batteries (PIBs) [212]; however, only a few phosphorus-based anode materials can reach limited success for K^+^ storage. Jin et al. [213] performed a comprehensive study of the electrochemical reactions of Li^+^, Na^+^, and K^+^ with BP. The lowest utilization of BP for K^+^ storage than for Na^+^ and Li^+^ has been revealed by ex situ X-ray absorption near-edge spectroscopy combined with theoretical calculation, which contributes to the highest formation energy and the lowest ion diffusion of the final potassiation product K_3_P, as compared with Li_3_P and Na_3_P. As a consequence, restricting the formation of K_3_P by limiting the discharge voltage could provide a gravimetric capacity of 1300 mAh g^−1^ which retains 600 mAh g^−1^ even after 50 cycles at 0.25 A g^−1^. To sum up, it is worth noting that all-solid-state batteries are becoming increasingly important due to the safety concerns. In the near future, further improvement of the performance of the secondary ion battery can be envisaged. Therefore, 2D phosphorene would have a broad application prospect in ion batteries as a positive electrode material for metal ion batteries.

### Li–S Batteries

Lithium–sulfur batteries (LSBs) have attracted enough attentions due to its specific capacity of 3861 and 1672 mAh g^−1^ for lithium and sulfur atoms, respectively. Meanwhile, the non-toxicity, natural abundance, and low price make it a potential alternative for current lithium-ion batteries [214–216]. The early development of lithium–sulfur technology can trace back to 1960s, and the high theoretical specific capacity (2600 Wh kg^−1^) is directly associated with combing two light elements as electrode materials. Lithium–sulfur batteries are composed of metallic lithium anode and octasulfur cathode [217]. During discharging process, metallic anode donates one electron per atom, while sulfur cathode gains two electrons for each sulfur atom [218]:

3 $$ {\text{Li}} \to {\text{Li}}^{ + } + e^{ - } $$

4 $$ {\text{S}} + 2e^{ - } \to {\text{S}}^{2 - } $$

5 $$ S_{8} + 16{\text{Li}} \to 8 {\text{Li}}_{2} {\text{S}} $$

**Fig. 13:**
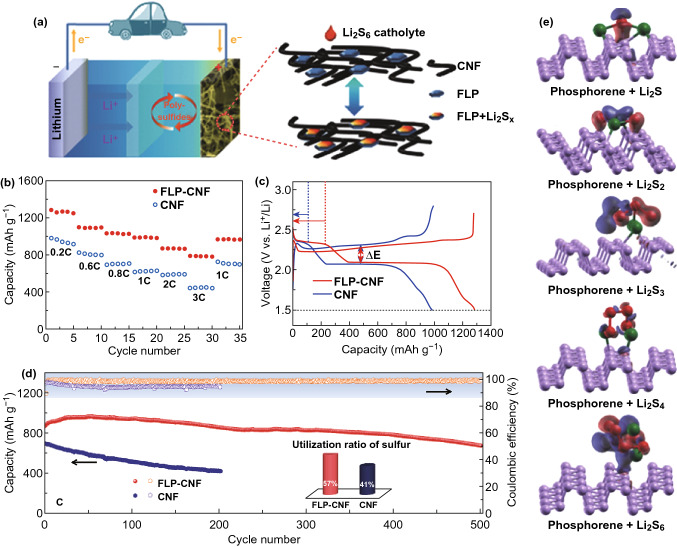
**a** Schematic of thin-layer black phosphorus/CNF hybrid materials as host for lithium polysulphide cathode. **b** Rate capabilities of CNF and 2D BP/CNF materials as cathode after 35 cycles at different current densities; **c** Galvanostatic charge–discharge voltage profiles of the first cycle at 0.2 C. **d** Cycling performance and coulombic efficiency of CNF and 2D BP/CNF materials after 500 cycles at a current density of 1 C. **e** Atom position and charge density plot for lithium polysulfide molecule interaction with phosphorene. (red-sulfur, green-lithium, violet-phosphorus); adapted with permission [225]. Copyright 2017 John Wiley and sons. (Color figure online)

The complete redox reaction provides an output voltage of 2.2 V [218]. However, several major limitations prohibit its further development. Firstly, the electrical and ionic conductivity of sulfur is very low. Secondly, the loss of sulfur in the electrolyte is inevitable. Thirdly, the sulfur shuttling interaction could occur between cathode and anode [219–223]. All of those shortcomings result in low reversibility and poor utilization of sulfur [224]. In order to tackle this issue, composite materials are proposed by combining sulfur with other materials, such as graphene, CNT, and phosphorene. Li et al. [225] introduced BP into a porous carbon nanofiber network to fabricate cathode. The comparison of battery performance can be seen in Fig. 13b–d. Obviously, the BP-embedded CNF cathode matrix shows greatly enhanced cycling and rating capability compared to the counterpart. The utilization of sulfur enhanced from 41 to 57% after the incorporation of BP. In order to better understand the contribution of BP, DFT calculations were performed to locate the atom position and charge density of BP layer and lithium polysulfide molecule (Fig. 13e). Furthermore, phosphorene can be applied as a modifier of membrane separator to capture and activate polysulfide, which can improve the subsequent cycling performance. Zhao et al. [226] theoretically investigated the absorption and diffusion mechanism of polysulfide on phosphorene. It is found that phosphorene exhibits absorption energy ranging from − 1 to 0.2 eV, indicating that it has a natural advantage of anchoring sulfur through the formation of strong phosphorus–sulfur covalent bond. By introducing phosphorene, the bandgap is reduced and the electrical conductivity is improved. Remarkable progress has been made in the application of 2D BP in LSBs. However, LSBs based on 2D BP are rarely reported since two-dimensional BP material is difficult to be prepared. The development of LSBs based on 2D BP still has a long period to go and needs the technical breakthrough for its efficient manufacturing. Lithium anode and electrolyte, besides 2D BP base cathode, are the other two key components to be noted for LSBs.

### Supercapacitors

**Fig. 14:**
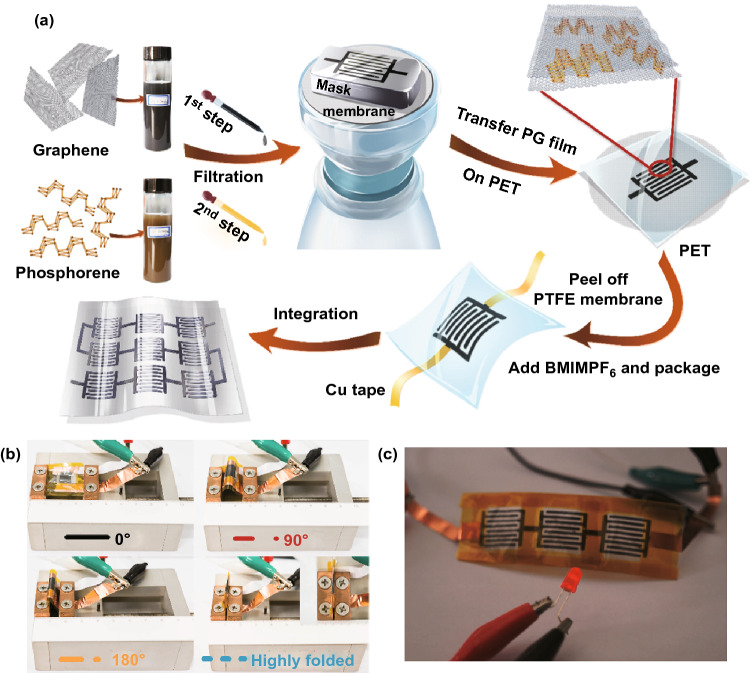
**a** Illustration of the fabrication of PG-MSCs, which includes the following steps: synthesis of graphene and phosphorene inks; step-by-step filtration of graphene and phosphorene in sequence with the assistance of an interdigital mask; dry transfer of PG hybrid film onto PET substrate; peeling off the PTFE membrane, drop-casting electrolyte, and device package; integration of serially interconnected MSC devices. **b** Flexibility comparison of PG-MSCs and PG-SSCs: Photographs of PG-MSCs at different bending states. **c** Photograph of three serial PG-MSCs used to power a light-emitting diode (LED). Adapted with permission [235], Copyright 2017 American Chemical Society

Supercapacitors are one of the major interests because of their long cycling stability, high power density, and fast current rate. Compared to secondary batteries, it can provide a much more stable power under high current density after long cycles [227–230]. It stores charge in electric double layer accumulated by ions at electrolyte/electrode interface, which enables energy recovery of heat-duty systems. In general, high-capacitance double-electrode capacitor requires large specific surface area. Therefore, 2D materials become one of the great candidates [231–234]. Nanocarbon materials have been previously studied, and the limited capacitance restrains its further applications. Meanwhile, theoretical calculations have indicated fast intercalation and diffusion of alkali ions owing to its large interlayer spacing, which could enhance electrochemical storage performance. As shown in Fig. 14, Xiao et al. [235] demonstrated flexible micro-SCs by combining the interdigital hybrid electrode pattern of 2D BP nanosheets and graphene (PG-micro-supercapacitors, MSCs). Remarkably, the device can be highly folded (Fig. 14b), indicating the superior flexibility and electrochemical stability. In addition, PG-MSCs exhibited a maximum volumetric energy density of 11.6 mWh cm^−3^, which can be ascribed to the synergistic effect between 2D BP and graphene. Moreover, the PG-MSCs can retain ~ 89.5% of its original capacitance after 2000 cycles. Notably, the as-prepared devices could light on a LED (Fig. 14c), illustrating the great potential for practical applications.

Although 2D BP possesses great potential for SCs electrode, its capacity needs to be enhanced to accommodate a wide range of challenging applications. Pore structure is one of the important factors affecting the properties of SC electrode, including pore size distribution, etc. The preparation of porous 2D BP is likely to be an effective way to improve its performance, i.e., constructing 3D BP by assembling 2D BP sheets. Thus, efficient electrolyte diffusion among them can be achieved. Meanwhile, a fast and continuous channel for electronic transmission can be achieved due to the intimate contact between 2D BP sheets. Besides, doping is an effective method to improve BP pseudocapacitance. With the passivation of the above BP, doping engineering may lead to high conductivity, and redox capacitances are introduced. Future optimization of manufacturing protocols will make 2D BP extremely attractive for energy devices in wearable electronics.

### Comparison with Other Two-Dimensional Non-carbon Materials for LIBs

In terms of chemical composition and structure, 2D non-carbon materials, which show potentials in energy storage applications, can be mainly divided into four different branches, including transition metal oxide materials (TMOs), elemental 2D materials (Xenes), TMDs, and MXenes. To thoroughly understand the performance of BP-based batteries, we also summarized some reported results as shown in Table 3. Similar to borophene [236], 2D BP can be considered in part of the MXenes. Besides, other MXene materials, such as stanene [237], also own buckled honeycomb structure and have been studied as anode materials due to its large spacing in crystal and interlayer distance. Mortazavi et al. [238] examined the energy storage performance of stanene, silicene, and germanene in LIBs through DFT analysis, and results show that the fully charged state of stanene and silicene is Li_0.97_Si and Li_0.97_Sn, respectively. The corresponding theoretical lithium storage capabilities are 226, 954, and 369 mAh g^−1^ for stanine, silicene, and germanene, respectively. Different from Xene materials, MXenes provide a lamellar-like alternating layers structure and it always contains an extra transition metal layer, which encapsulates carbon/nitrogen elemental layer [11, 239–243]. Tang et al. [244] studied the Ti_3_C_2_ via DFT calculation and found a low lithium atom diffusion barrier and high theoretical capacity (320 mAh g^−1^) in LIBs. Byeon et al. [245] fabricated Ti_3_C_2_T_x_/CNT composite materials, and it can deliver an excellent cycling performance with a high specific capacity of 428 mA  g^−1^ under current density at 0.5 C after 300 cycles.

**Table 3:** Summary of energy storage performances of 2D BP and other 2D materials

Electrode materials	Battery type	Battery performance	References
phosphorene	LIBs	432.79 mAh g^−1^ (theoretical)	[149]
Stanene	LIBs	226 mAh g^−1^ (theoretical)	[204]
Silicene	LIBs	954 mAh g^−1^ (theoretical)	[204]
Germanene	LIBs	369 mAh g^−1^ (theoretical)	[204]
Ti_3_C_2_	LIBs	320 mAh g^−1^ (theoretical)	[210]
MoS_2_	LIBs	1290 mAh g^−1^ (theoretical)	[212–215]
WS_2_	LIBs	433 mAh g^−1^ (theoretical)	[219]
V_2_O_5_	LIBs	294 mAh g^−1^ (theoretical)	[221–225]
2D BP	LIBs	210 mAh g^−1^	[154]
2D BP/graphene	LIBs	820 and 920 mAh g^−1^ at 100 mA g^−1^	[124, 154]
Ti_3_C_2_T_x_/CNT	LIBs	428 mAh g^−1^ after 214 mA g^−1^	[211]
MoS_2_/C	LIBs	1150 mAh g^−1^	[217]
C@TiO_2_/MoS_2_	LIBs	1072 mAh g^−1^ at 1A g^−1^	[218]
WS_2_/SWCNT	LIBs	861 mAh g^−1^ at 100 mA g^−1^	[219]
WS_2_/CNF	LIBs	545 mAh g^−1^ at 500 mA g^−1^	[220]
WS_2_/GCNF	LIBs	1069 mAh g^−1^ at 100 mA g^−1^	[220]
V_2_O_5_	LIBs	211 mAh g^−1^ at 150 mA g^−1^	[226]
Phosphorene	SIBs	865 mAh g^−1^ when Na_2_P is formed	[169]
Phosphorene/graphene	SIBs	2440 mAh g^−1^ at 48.8 mA g^−1^	[175]
2D BP	SIBs	1968 mAh g^−1^ at 100 mA g^−1^	[119]
2D BP-PANI	SIBs	~ 250 mA h g^−1^ under 0.5 A g^−1^	[200]
2D BP-MXene	SIBs	535 mAh g^−1^ at 0.1 A g^−1^	[201]
2D BP	MIBs	2205 mAh g^−1^ (theoretical)	[174]
2D BP/graphite	PIBs	1300 mAh g^−1^ at 0.75 A g^−1^	[212]
Few-layer phosphorene/CNF	LSBs	1262 mAh g^−1^ at 335 mA g^−1^	[225]
2D BP	SC	13.75 F cm^−3^ at 0.01 V s^−1^	[200]
2D BP film	SC	17.78 F cm^−3^ at 0.005 V s^−1^ and 1.43 F cm^−3^ at 10 V s^−1^	[229]
2D BP/G film	SC	37.5 F cm^−3^ at 0.005 V s^−1^	[235]
2D BP/polyaniline	SC	354 F g^−1^ at 0.3 A g^−1^	[230]

TMDs have recently been reported as promising alternative for conventional anode materials. It has a sandwich-like structure, which consists of two chalcogen layers and one transition metal layer in the middle [246–249]. Two main phases, 1T and 2H, exist among TMD materials with chalcogen atoms localized in either octahedral or trigonal coordination of transition metal atoms. Moreover, 2H phase of TMDs is generally considered as the most thermodynamically stable state. MoS_2_ is one of the representatives from TMD materials, and it can deliver a theoretical specific capacity up to 1290 mAh g^−1^, which makes it a competitive candidate for LIBs anode materials [250]. Zhao et al. [251] prepared MoS_2_/C composite materials as anode, and it could provide a specific capacity of 1150 mAh g^−1^ with retention ~ 100% after 100 cycles with the MoS_2_ mass ratio of 47%. Wang et al. [252] demonstrated a self-supported C@TiO_2_/MoS_2_ composite anode material with a specific capacity of 1072 mAh g^−1^ after 1000 cycles at 1 A g^−1^. WS_2_ is another representative of TMD materials with a high theoretical specific capacity of 433 mAh g^−1^. Liu et al. [253] developed WS_2_/SWCNT material, and it delivered a specific capacity of 861 mAh g^−1^ at 100 mA g^−1^ after 50 cycles. WS_2_/CNF was also prepared and examined, which retained a good cycling performance. Discharge capacity of WS_2_/CNF remained at 545 mAh g^−1^ after 800 cycles under current density of 500 mA g^−1^. Zhang et al. [249] promoted a novel technique to prepare WS_2_/GCNF composite as anode material. The retention maintained as high as 95% with a specific capacity of 1069 mAh g^−1^ after 100 cycles at 100 mA g^−1^.

V_2_O_5_, as part of the TMOs, has layered crystal structure accompanied with excellent flexibility. Each layer of V_2_O_5_ is constructed with distorted trigonal bipyramidal polyhedral, which forms zigzag double chains and cross-linking by sharing edges and corners along (001) and (100) directions. It is confident that it could be used as anode materials in LIBs with a theoretical value of 294 mAh g^−1^ [202, 254–257]. Kong et al. [258] produced a CNT/V_2_O_5_, where V_2_O_5_ was enclosed by carbon nanotubes, with 91.7% capacity retention. After 200 cycles, the specific capacity maintained at 211 mAh g^−1^ under a current density of 150 mAh g^−1^.

Similar to 2D BP, those 2D materials also experience some serious defects like self-stacking, causing high initial irreversible capacity as well as low coulombic efficiency as compared to bulk materials, and high SSA leading to unnecessary side effects, including electrolyte breakdown especially on the anode side. The products of electrolyte decomposition (usually lithium carbonate and organic components) are easy to dissolve in the charging process, making the electrolyte deterioration and leading to poor cycle life, and safety problems. But different from 2D BP, those 2D materials usually also have great performance as cathodes for batteries. In terms of the layered structure, two-dimensional nanomaterials are easy to assemble into flexible electronics, especially for those graphene and graphene-based composites, which cater for the development of portable electronic devices. There is an urgent need, while it has a significant challenge to improve production rate and control the precise structure of 2D nanomaterials.

## Challenges and Future Perspectives

2D materials have attracted much attention in the field of battery due to their large surface area, high carrier mobility, and fast response, which has been widely applied in the ultrafast laser generation, optical switching and modulators, optoelectronics devices and biosensor and biotherapy. This review has comprehensively presented the current progress of 2D BP materials with their synthesis, properties, and energy storage applications. In summary, the attractive properties (e.g., high mechanical strength, good ion conductivity) of thin-layer 2D BP allow it to be further studied for future energy storage applications. However, the research on 2D BP is still at the infancy stage with many potentials remain to be unexploited. Some major challenges as well as opportunities have been identified and widely accepted.

First of all, the structural morphology of 2D BP produced by different preparation methods is usually different, so its energy storage performance could be also varied. The intrinsic relationship between the preparation method, structure and morphology, and energy storage performance of 2D BP needs to be explored. Secondly, restacking of thin-layer BP can hardly be avoided when applied as anode materials, which can cause large volume expansion after cyclic application. Therefore, 2D BP composite materials can be investigated in future to improve its energy storage performance by optimizing its composite like structure, using highly conductive carbon material as a carrier. Thirdly, 2D BP can degrade in the presence of moisture, visible light, and oxygen, which could significantly restrict its practical applications. Therefore, finding a reliable technique (e.g., encapsulation) to resolve this issue without deteriorating its performance is in demand. In addition, it is still not clear how the electrolyte and binder affect the energy storage performance when BP and phosphorene are employed as anode for secondary batteries. The last but not the least, there are only some researches on BP or phosphorene in supercapacitors, and its reaction mechanism in different oxides needs to be further explored. There are many deficiencies in a single material system. Composite electrode materials should be the focus of future research. In addition, the construction of three-dimensional BP structure is an effective approach to further enhance the properties of 2D BP materials. 3D hierarchical structure has the following advantages: I) The greatly tunable pore structure can increase specific surface area and promote the diffusion of ion. II) 3D network with high conductivity provides fast electronic transmission; III) the 3D interconnection structure can buffer volume changes during charge and discharge cycles.

On the other hand, traditional electrochemical characterization techniques, such as cyclic voltammetry, constant current intermittent titration, and electrochemical impedance spectroscopy, can reveal the performance of the hybrid electrode, but it is hard to detect the intrinsic properties of the active material during ion insertion. 2D BP nanosheets with high electrochemical activity could act as a promising material platform to investigate those properties at the micro- and nanoscale, such as their electron and heat transfer properties, optical properties, as well as charge transfer properties, due to the possibility of processing into nanoscale equipment, and the possibility of in situ observation. Therefore, more researches are needed to understand the potential mechanisms of ion-intercalation-induced 2D BP behavior change, especially the electron structure and charge transfer characteristics.

Simultaneously, many other new 2D materials have been developed in the last two decades. Except from BP, some other layered materials (e.g., As, Sb, Bi) also own a similar puckered structure that is highly in-plane anisotropic. Most of them exhibit tunable bandgaps and high charge carrier mobilities. Recently, 2D BP analogies have made a big progress both theoretically and experimentally. In 2015, monolayer arsenene and antimonene have been theoretically proved to possess stable chemical structure. The bandgap is calculated to be 2.49 and 2.28 eV (blue light spectrum), respectively. Then, antimonene was successfully prepared in 2016. Meanwhile, bismuthene, which is the heaviest element in the family of VA group, is also attracting a lot of attention. Theoretically, bismuthene has strong quantum spin Hall effect (QSH) with relatively large bandgap. For the future research, those phosphorene analogies can be helpful for developing energy storage devices, such as hybridizing them with thin-layer BP as composite materials. Due to the diversity of phosphorene and its analogies, the demanding of high energy storage applications can be achieved by manipulating the chemical structure and physical composition of the composite materials. Therefore, the future research in 2D BP analogies and their combinations with thin-layer BP can be further explored.
